# Faculty of Bellevue Medical College and Prof. James P. White

**Published:** 1871-07

**Authors:** Isaac E. Taylor, Austin Flint

**Affiliations:** President; Secretary


### Faculty of Bellevue Medical College and Prof. James P. White.

We to notice the following resolutions were offered to Prof. James P. White.

" The *Faculty of the Bellevue Hospital Medical College*, desirous ot expressing their sentiments in relation to the services of Professor James P. White in behalf of the College during the session of 1870-71, unanimously adopted, April 4th, 1871, the following resolutions:—

*Resolved*, That the Faculty were peculiarly favored in being able to secure the services of so eminent a teacher and practitioner as Professor James P. White, when the College was deprived, by illness, of the services of their late lamented colleague, Professor George T. Elliott.

*Resolved*, That the lectures given by Professor White in the Bellevue Hospital Medical College were characterized by great learning, the practical knowledge derived from large experience, zealous exertions to render his instructions as useful as possible, and an efficiency showing peculiar ability and qualifications as a public teacher. These characteristics were fully appreciated by the class, who received his lectures with gratitude and enthusiasm.

*Resolved*, That in refusing to receive compensation for his lectures, relinquishing the fees to Prof. Elliott, Professor White exemplified a spirit of sympathy and generosity which is deserving of admiration.

*Resolved*, That the Faculty will ever cherish the remembrance of their pleasant social intercourse with Professor White during his residence in New York, and they most cordially tender wishes for his welfare, and for a long duration of his active usefulness.

*Resolved*, That a copy of these resolutions be properly engrossed, attested, and bearing the College Seal, be transmitted to Professor White.

ISAAC E. TAYLOR, M. D., *President.*
AUSTIN FLINT, Jr., M. D., *Secretary.*

